# Dioscin relieves endotoxemia induced acute neuro-inflammation and protect neurogenesis via improving 5-HT metabolism

**DOI:** 10.1038/srep40035

**Published:** 2017-01-06

**Authors:** Rui Yang, Wei Chen, Ye Lu, Yingke Li, Hongli Du, Songyan Gao, Xin Dong, Hongbin Yuan

**Affiliations:** 1 Department of Anesthesiology, Changzheng Hospital, Second Military Medical University, Shanghai 200003, China; 2 Changhai Hospital, Second Military Medical University, Shanghai 200433, China; 3 School of Pharmacy, Second Military Medical University, Shanghai 200433, China

## Abstract

Sepsis, in addition to causing fatality, is an independent risk factor for cognitive impairment among sepsis survivors. The pathologic mechanism of endotoxemia induced acute neuro-inflammation still has not been fully understood. For the first time, we found the disruption of neurotransmitters 5-HT, impaired neurogenesis and activation of astrocytes coupled with concomitant neuro-inflammation were the potential pathogenesis of endotoxemia induced acute neuro-inflammation in sepsis survivors. In addition, dioscin a natural steroidal saponin isolated from Chinese medicinal herbs, enhanced the serotonergic system and produced anti-depressant effect by enhancing 5-HT levels in hippocampus. What is more, this finding was verified by metabolic analyses of hippocampus, indicating 5-HT related metabolic pathway was involved in the pathogenesis of endotoxemia induced acute neuro-inflammation. Moreover, neuro-inflammation and neurogenesis within hippocampus were indexed using quantitative immunofluorescence analysis of GFAP DCX and Ki67, as well as real-time RT-PCR analysis of some gene expression levels in hippocampus. Our *in vivo and in vitro studies* show dioscin protects hippocampus from endotoxemia induced cascade neuro-inflammation through neurotransmitter 5-HT and HMGB-1/TLR4 signaling pathway, which accounts for the dioscin therapeutic effect in behavioral tests. Therefore, the current findings suggest that dioscin could be a potential approach for the therapy of endotoxemia induced acute neuro-inflammation.

Sepsis is characterized by a cascade of amplified systemic inflammation resulting in a lot of critical clinical implications, including central nervous system disorders such as neuronal degeneration and memory impairment, which occurs in 8 to 70% of septic patients^1^,^2^,^3^,^4^. Survivors of sepsis usually have a long-term of cognitive dysfunction over the rest of their lives after being discharged from hospitals^5^.

Based on clinical evidences, in addition to being a cause of fatality, sepsis, especially sever sepsis, is an independent risk factor for cognitive impairment and functional disability among sepsis survivors^6^. Although there are many hypotheses, including oxidative stress injury, neuro-inflammation, microglia and astrocyte activation, neurotransmission imbalance, and neuronal apoptosis have been proposed but the precise pathologic mechanisms of endotoxemia induced acute neuro-inflammation and cognitive impairment are still not fully understood^7^,^8^,^9^.

Traditional Chinese Medicine, a great treasure in China for thousands of years, has drawn great interest across the world for its efficacy in the treatment of many diseases^10^. Dioscin, is a natural steroidal saponin isolated from certain Chinese medicinal herbs, such as Dioscoreanipponica Makino and Dioscoreazingiberensis C. Recently, dioscin was reported to possess anti-inflammation, anti-tumor, and anti-hyperlipidemia activities^11^,^12^ and previous studies have proved that dioscin regulates neuro-inflammation through HMGB-1/TLR4 signaling pathway and ameliorates cerebral ischemia/reperfusion injury^13^. Therefore, we wanted to investigate whether dioscin also has neuro-protective and inflammatory regulatory effects against endotoxemia induced acute neuro-inflammation, and its related mechanism.

5-HT (5-hydroxytryptamin), one of the monoamine transmitters, is a well-known antidepressant agent^14^. Previous reports^15^ showed that 5-HT plays an important role in serotoninergic system and is involved in the treatment of depression. Therefore, we hypothesized that endotoxemia induced impairment of 5-HT neuron transmitter metabolism and activation of neuron immune cells are involved in the pathophysiologic process of endotoxemia induced acute neuro-inflammation and cognitive impairement^16^, and dioscin ameliorates endotoxemia induced acute neuro-inflammation and accommodates neuron inflammation through HMGB-1/TLR4 signaling pathway.

## Results

### LPS intra-peritoneal injection induced depressive-like behavior in animals and dioscin treatment improved animal behavior in Open Field Test (OFT) and Forced Swimming Test (FST)

**Figure 1:**
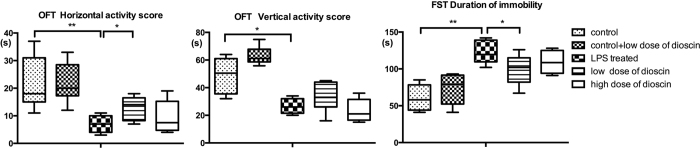
LPS intra-peritoneal injection induced anxiety-like behavior in animals and dioscin treatment improved animal behavior in open field test and forced swimming test. Open field tests were performed twenty-four hours after LPS injection. Vertical activity score and horizontal activity score in OFT. Duration of immobility was recorded in 6-min FST (c) (n = 6 per group). Dioscin was given by oral gavage at a dose of 25 (low dose) and 37.5 mg/kg (high dose), respectively, once daily for three days before LPS injection. The results are expressed as the mean ± SEM from eight mice per group. *P < 0.05, **P < 0.01.

In the OFT, the vertical activity score was defined as the number animal crossed the lines which indicates the ability to explore a new environment. Compared with control group, mice with LPS treatment showed a significant decrease in exploration. LPS treatment successfully induced a depressive-like behavior (Fig. 1). The vertical activity score, calculated by the times of verticality, had a statistical significant difference between control group and LPS treated group as well as LPS treated group and low does dioscin group. Compared with mice in control group, animals treated with LPS exhibited apathetic behavior. Immobility was regarded as depressive-like behavior in FST and mice in LPS treated group appeared less active compared with control group. Meanwhile, dioscin administration at 25 mg/kg was able to significantly reduce the immobility time. Therefore, dioscin at 25 mg/kg improved the mouse behavior and had an antidepressant-like effect in both OFT and FST. However, based on the behavior testing results, the high dose dioscin treated group (37.5 mg/kg) didn’t show a protective effect on endotoxemia induced acute cognitive impairement. In that regard, we chose 25 mg/kg as dioscin dosage for the rest of the study.

### 5-HT level was greatly decreased in endotoxemia pathological process and dioscin exerted its antidepressant effect by increasing 5-HT levels

**Figure 2:**
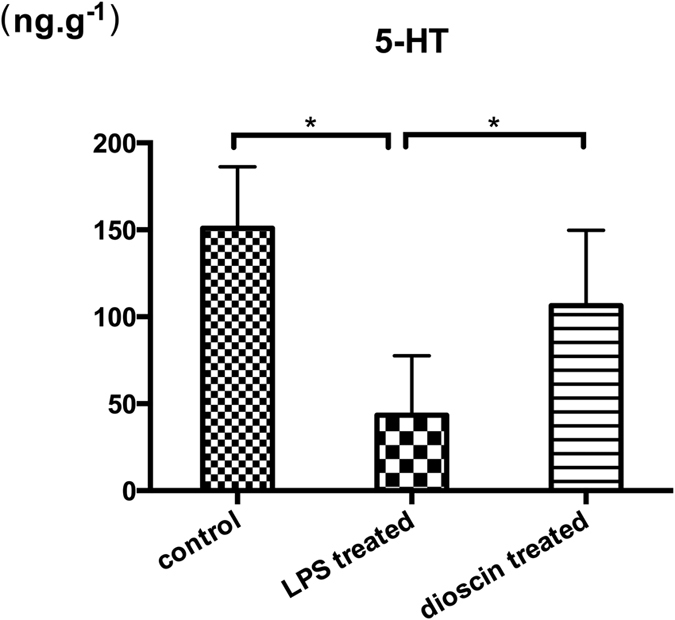
5-HT level was markedly decreased in endotoxemia pathological process and dioscin exerted its antidepressant effect by increasing 5-HT levels. Levels of 5-HT in hippocampus in mice from different groups were analyzed by HPLC. The amount of 5-HT in each sample calculated according to the area under curve. In dioscin treated group, mice were administrated with dioscin at a dose of 25 mg/kg. The results are expressed as the mean ± SEM from eight mice per group. *P < 0.05.

5-HT, one of the monoamine transmitters, is well-known to be involved in the effects of antidepressant^17^. Concentration of 5-HT in mice hippocampus from control, LPS treated, and dioscin treated groups were determined by high-performance liquid chromatography (HPLC). As shown in Fig. 2, in LPS treated group, the level of 5-HT in hippocampus was greatly decreased in comparison with control group. And dioscin treatment (25 mg/kg) significantly increased 5-HT level in hippocampus (p < 0.05), indicating that dioscin can enhance the serotonergic system and exert anti-depressant effect by increasing 5-HT levels.

### Dioscin regulated neuro-inflammation via HMGB-1/TLR4 signaling pathway

**Figure 3:**
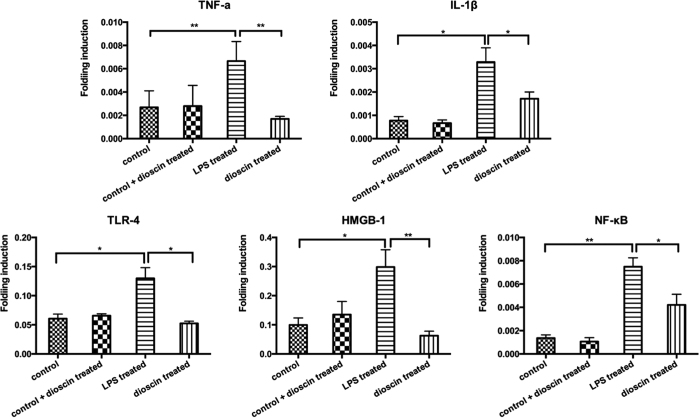
The LPS treated group showed dramatic increases in the levels of inflammatory mediators, including TNF-α and IL-1β as well as their upstream signal molecules such as HMGB1, TLR4 and NF-κB. Q-RT-PCR assay of TNF-α, IL-1β, HMGB1, TLR4 and NF-kB mRNA expression in hippocampus from the control, LPS treated and LPS + dioscin treated groups. Error bars represent mean ± SEM. *p < 0.05.

Previous studies have demonstrated: Tryptophan and its derivatives 5-HT have a protective effect on neuro-inflammation^18^. Thus, based on the changes in hippocampus 5-HT level, we wondered whether classic HMGB-1/TLR4 related inflammation pathway participates in the pathological process of endotoxemia induced acute neuro-inflammation. To our satisfaction, the LPS treated group showed dramatic increases in the levels of inflammatory mediators, including TNF-α andIL-1β, as well as their upstream signal molecules like HMGB1, TLR4 and NF-κB (Fig. 3), compared to the control group, indicating that they may be responsible for the activation of neuro-inflammation and damage of neurons in endotoxemia induced impairment in the central nerve system. After dioscin treatment, the HMGB-1/TLR4 signaling had a tendency to be rectified to normal, which demonstrated that maybe dioscin could regulate neuro-inflammation cascade through HMGB-1/TLR4 signaling pathway. By integrating different experimental platforms, including metabonomics and classic molecular biological technique, we found HMGB-1/TLR4 inflammation signaling pathway may contribute to endotoxemia induced acute neuro-inflammation, but the role of this classic signaling pathway in neuro-inflammation process needs further investigation.

**Figure 4:**
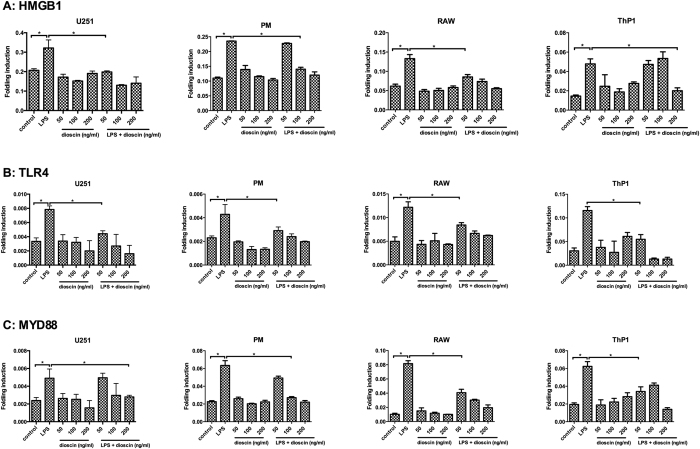
The LPS treatment increased the transcript levels of some signal molecules including HMGB1, TLR4 and Myd88, and dioscin was able to rectified the overexpressed mRNA in immune cells and human glioma cell lines. The Q-RT-PCR assay of HMGB1 (**A**), Myd88 (**B**) and TLR4 (**C**) mRNA expression in mouse primary peritoneal macrophages (PM), human U251 glioma cell line RAW264.7 cell line and Thp1 monocyte cell line from the control, LPS treated diefferent concentrations of dioscin (50, 100, 200 ng/ml) treated and LPS + dioscin (50, 100, 200 ng/ml) treated groups. Error bars represent mean ± SEM. *p < 0.05.

In addition, we chose immune cell and neuroglia cell lines for to elucidate the mechanisms of dioscin’s neuro-protective effects. As shown in Fig. 4, the mRNA levels of HMGB1, Myd88 and TLR4 were significantly increased in mouse primary peritoneal macrophages (PM), mouse primary peritoneal macrophages (PM), neuroglia cell line U251 from human glioma, macrophages cell line RAW264.7 and monocyte cell line THP1 from LPS group upon LPS stimulation, while different concentrations of dioscin treatment showed its protective effect by rectifying HMGB-1/TLR4/Myd88 signaling to normal, consistent with our *in vivo* results. Moreover, the CCK8 cell proliferation assay (Fig. S1) and apoptosis analysis by flow cytometry (Fig. S2) demonstrated dioscin at even high concentrations was safe.

### Dioscin alleviated astrocyte activation and protected neurogenesis in endotoxemia

**Figure 5:**
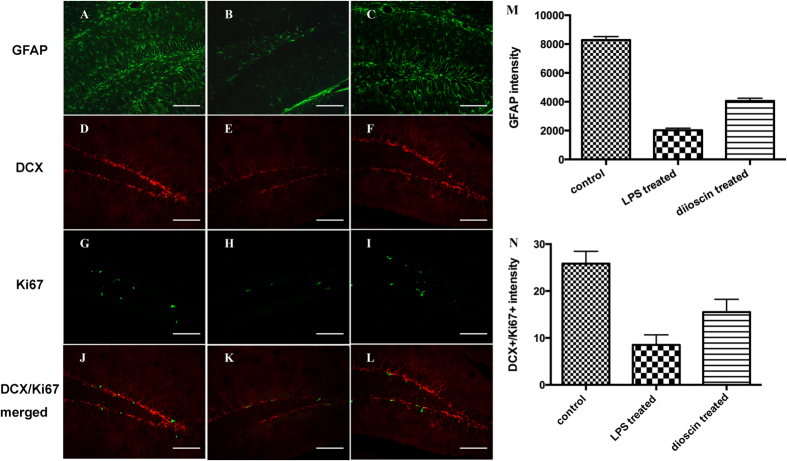
Dioscin alleviated astrocyte activation and had a protective effect on neurogenesis from endotoxemia induced acute neuro-inflammation and cognitive impairment. Immunofluorescence staining for GFAP (green) (**A**–**C**), DCX (red) (**D**–**F**) and Ki67 (green) (**G**–**H**) in hippocampus of mice from control, LPS treated and LPS + 25 mg/kg dioscin treated groups were constructed. Co-localization of different markers is visualized in composite images for double immunostainings showing Ki67 and DCX (**J**–**L**). (Scale bar, 100 μm) The mean number of GFAP^+^ and DCX^+^/Ki67^+^ neurons per section in the hippocampus from the control, LPS treated and LPS + 25 mg/kg dioscin treated groups were calculated (**M**–**N**). The data are presented as the mean ± standard error (n = 6/group; * p < 0.05).

To gain insight into neuro-inflammation and neuron damage in endotoxemia induced acute cognitive impairment, we further assessed astrocyte responses and neurogenesis in hippocampus. Glial fibrillary acidic protein (GFAP) is a marker for astrocytes^19^. GFAP immune-reactive astrocytes, labeled with green fluorescence, appeared normal, with branches uniformly distributed in the hippocampus of control mice (Fig. 5A). Twenty-four hours after LPS injection, a great number of GFAP immune-reactive astrocytes with thick processes were observed in hippocampus (Fig. 5B). Apparently, there was an acute activation of astrocytes in the hippocampus of mice from LPS treated group. With dioscin treatment, the number of GFAP immuno-reactive astrocyte decreased greatly (Fig. 5C), indicating that dioscin may have a potential role in the regulation of neuro-inflammation and treatment of endotoxemia induced acute neuro-inflammation.

Doublecortin (DCX), a marker for neuronal progenitors and immature neurons, is expressed in the early stage of new neurons^20^, and Ki67, an endogenous protein expressed in actively dividing cells from G1-phase through M-phase, is used as a marker of cell proliferation^21^. We observed the numbers of DCX and Ki67-positive cells (Ki67: G and H) were decreased in the LPS treated group (Fig. 5D,E), indicating LPS treatment leads to a loss of adult neurogenesis, and quantitative analysis of immunoreactivity of these proteins revealed that dioscin treatment was able to reverse the LPS induced endotoxemia damage and had a protective effect on neurogenesis from neuro-inflammation, which is consistent with the results of behavior test.

### 5-HT related metabolic pathway was involved in pathogenesis of endotoxemia induced acute neuro-inflammation

**Figure 6:**
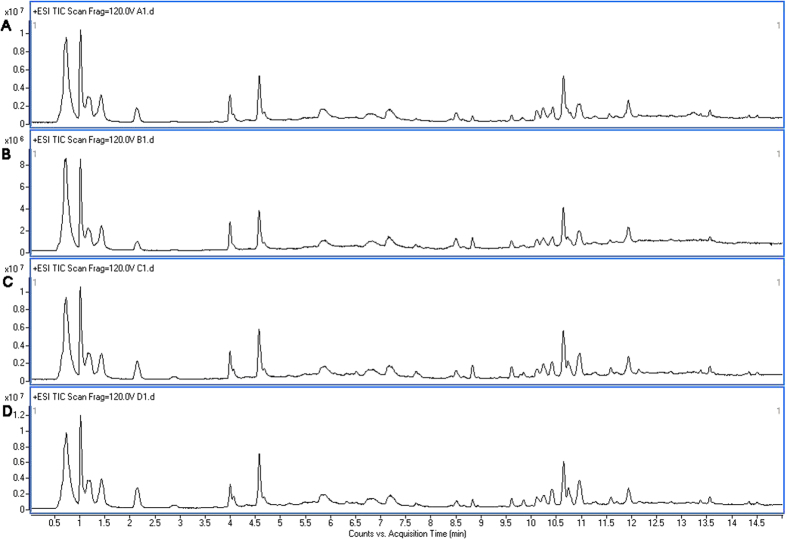
Representative base peak chromatograms obtained in ESI positive ion from control group (**A**), dioscin treated group (**B**), LPS-treated group (**C**) and LPS + dioscin treated group (**D**). By visual inspection, the spectra of the four groups showed a very similar profile, which indicated that certain target metabolites were affected and played a role in crystal model group and then they were rectified by dioscin treatment.

**Figure 7:**
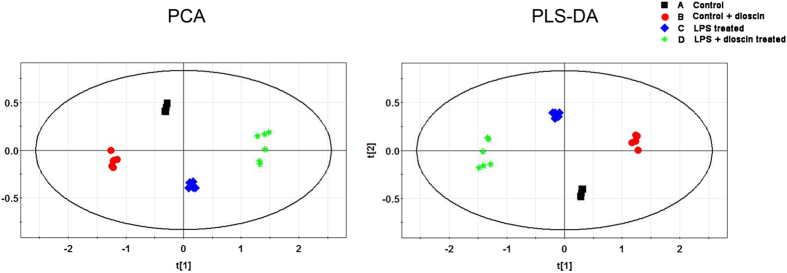
Chemometric analysis by principle component analysis (PCA) score plot and partial least squares discriminate analysis (PLS-DA) score plot and loadings plot derived from UHPLC-MS data of hippocampus samples from the control group (**A**), dioscin treated group (**B**), LPS-treated group (**C**), and LPS + dioscin treated group (**D**) was shown. Overall, in PCA score plot discrimination of all three groups was shown in the score plot while the LPS + dioscin treated group was close to the saline control group. From the PLS-DA score plot, all of the three groups were separated from each other. Additionally, the loading plot showed which metabolites contributed the most to the discrimination.

Since brain 5-HT is an important member of monoamine neurotransmitters and the imbalance of 5-HT participates in many metabolic disorders in the central nerve system, we wondered whether there were metabolic disorders in hippocampus during pathological process of endotoxemia induced acute neuro-inflammation. In order to explore the metabolic change of hippocampus and mechanism of the protective effects of dioscin, ultra-high performance liquid chromatography-quadrupole time-of-flight mass spectrometry (UHPLC-Q-TOF/MS) analysis was performed both in positive mode and negative mode. As shown in the electro spray ionization (ESI) (Fig. 6), all of the three spectra were in similar to each other, which was illustrated in Fig. 6A (control group), Fig. 6B (dioscin treated group), Fig. 6C (LPS treated group) and Fig. 6D (LPS + dioscin treated group) in positive mode. After preprocessed data set by electro spray ionization, principle component analysis (PCA) was performed to extract systematic variations as well as to visualize the covariance and corresponding among the control group, the LPS treated group and LPS + dioscin treated group (Fig. 7). Furthermore, partial least squares discriminate analysis (PLS-DA) was applied to screen the potential biomarkers and the score plot (Fig. 7) demonstrated that LPS treated group was obviously separated from control group, while LPS + diosicn treated group was similar to control group, which further proved that dioscin could rectify the metabolic deviation and had protective effect against endotoxemia induced injury in central nerve system (Fig. 7). Meanwhile, results of the permutation test showed that no over-fitting was observed. Each VIP value of the metabolite calculated by SIMCA P11.0 software signified the influence of each metabolite on the classification.

**Figure 8:**
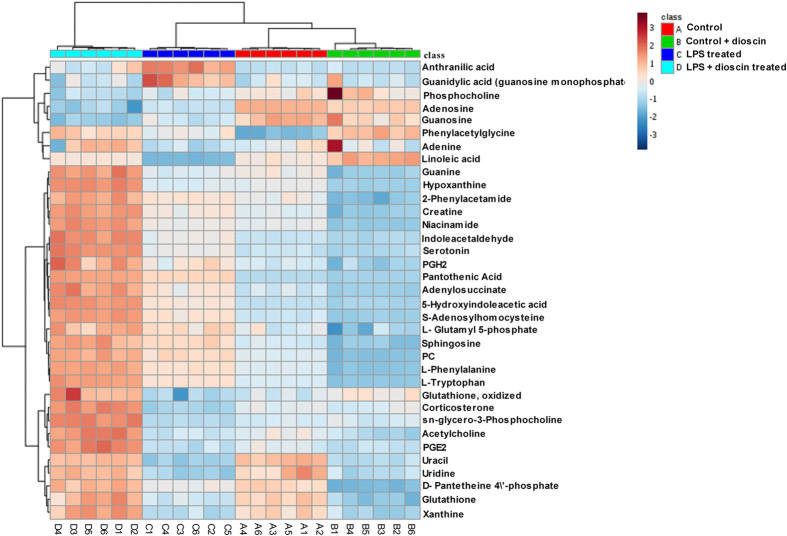
Heat map based on the normalized quantities of the 34 most significantly different metabolites in the control group (A1–A6), dioscin treated group (B1–B6), LPS-treated group (C1–C6) and LPS + dioscin treated group (D1–D6), which showed altered concentration of differential metabolites by dioscin treatment.

The mass-based identification of the potential metabolic biomarkers was developed through online databases including Metlin database (http://www.metlin.scripps.edu) and Human Metabolome Database (http://www.hmdb.ca/). Thirty-four metabolites (Table 1) were finally selected as potential biomarkers of endotoxemia induced acute neuro-inflammation whose levels were significantly different between control group and LPS treated group, as well as between LPS treated group and LPS+25 mg/kg dioscin treated group (p < 0.05). Among them, metabolites such as 5-hydroxyindoleacetic acid, L-tryptophan, and indoleacetaldehyde are products of neuro-transmitter 5-HT and have an influence on the tryptophan related metabolism, consistent with the results from neuro-transmitter analyses. To further characterize the therapeutic effects of dioscin, a heat map of the 34 important metabolites was performed by Metabo Analyst platform (http://metaboanalyst.ca) to visualize the variation in the relative intensity of each metabolite among control group, dioscin treated group, LPS treated group and LPS + dioscin treated group (Fig. 8), which showed the altered concentration of differential metabolites by dioscin treatment.

**Figure 9:**
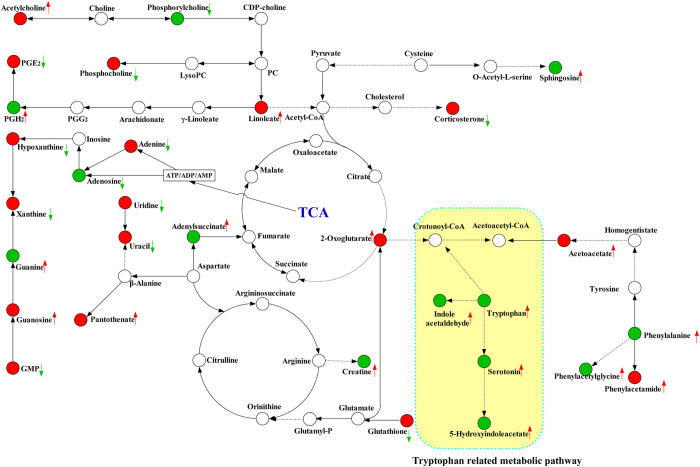
A metabolic pathway map showed the changed metabolites and their related pathway in LPS treated group or after dioscin treatment. The green arrow represents, in comparison with control group, metabolites decreased in LPS treated group. The red arrow represents, in comparison control group, metabolites increased in LPS treated group. The green circle represents, in comparison with LPS treated group, dioscin treatment did not rectify the change of metabolites. The red circle represents, in comparison with LPS treated group, dioscin treatment can effectively rectify the change the tendency of metabolites.

To further characterize the metabolic disorder in the process of endotoxemia induced acute neuro-inflammation and cognitive impairment as well as the therapeutic effect of dioscin, a metabolic picture was drawn by the Smartdraw software. Figure 9 shows a metabolic pathway, including related energy metabolism and amino acids metabolism, especially tryptophan metabolism consistent with the results from neuro-transmitter analyses. In addition, dioscin also had a corrective effect on these metabolic disorders, indicating the protective effects of dioscin in behavior tests and 5-HT metabolism.

## Materials and Methods

### Animal experiment and sample collection

Thirty-two male C57BL/6 mice (two month-old) were purchased from the Shanghai SLAC laboratory Animal Co., Ltd. All animal experiments were approved by the Animal Ethics Committee of Changzheng Hospital and were performed according to the Guide for Care and Use of Laboratory Animals published by the US National Institutes of Health (NIH publication no.85-23, revised 1996). Mice were housed under a 12-h light/dark cycle (lights on at 7 AM–7 PM) in a temperature-controlled room at 24±1 °C (What is this symbol on the left?) and had free access to food and water. Mice were randomly divided into four groups: control, LPS (Lipopolysaccharides from Escherichia coil O111: B4, Sigma-Aldrich, St Louis, MO) treated, and LPS with a low, and a high dosage of dioscin treated group (six mice in each group) Saline and LPS injections were freshly prepared for each treatment. First, mice in dioscin (Yuanye Biotech, China) group were administered by oral gavage at the dose of 25 and 37.5 mg/kg, respectively, once daily for 3 days, and mice in control and LPS group only received equal volume of saline. For *in vitro* experiment dioscin was added to the serum free medium after it was dissolved in DMSO and the final concentration of dimethyl sulfoxide (DMSO) was less than 0.1%. Because previous studies have shown that LPS resulted in a long-lasting neuro-inflammation and persistent changes in neurophysiological, cognitive and behavioral parameters^4^,^22^,^23^,^24^, we administered mice with endotoxemia by an intra-peritoneal injection of LPS dissolved in saline at a dose of 10 mg/kg 24 hours before behavioral tests, while mice from control group received an equal volume of saline.

### Cell culture and *in vivo* experiments

Primary peritoneal macrophages (PM) were collected from mice after an intraperitoneal injection of 1 mL of 3% thioglycolate (Sigma-Aldrich). All cell lines including RAW264.7, human U251 glioma cells and ThP1 monocyte cell line were purchased from the Institute of Biochemistry Cell Biology (Shanghai, China). All cells were cultured in PRMI and DMEM medium supplemented with 10% fetal bovine serum and incubated in a humidified atmosphere of 5% CO_2_ and 95% O_2_ at 37 °C. LPS was prepared in ultrapure water and make a work concentration of 500 ng/ml and dioscin was diluted in different concentrations of 50, 100, and 200 ng/ml in medium. Dioscin at different concentrations was directly applied to cell cultures for 24 hours before challenged with LPS. 24 hours later treated cells were lysed with TRIzol reagent for RNA extraction and Q-RT-PCR analyses.

### Behavioral tests

All behavioral tests were performed in a sound proof room and by a lab technician who was blind to experiment design. Twenty-four hours after the septic LPS/saline injection, mice were subjected to OFT and FST. For OFT, mice were gently placed in the center arena (60 × 60 × 35 cm^3^) for 5 min. In order to assess animal’s mobility, behavior and emotion changes, we recorded horizontal activity scores by the number of mice crossed the lines and vertical activity scores by the times of front paws off the ground. In FST, based on the method of Porsolt *et al*.^25^, mice were placed individually in an open clear glass container (12 cm diameter, 40 cm tall) with water at a temperature of 24 ± 1 °C and a depth of 12 cm. Immobility in the animals was regarded as depressive-like behavior, defined as the absence of movement or struggles to survive, such as leg kicking to stay afloat and keeping head above the water. The total duration of immobility was recorded in the final 4 minutes in a 6-min test. At the end of the test, each mouse was removed from water and placed in a warming cage to dry.

### Tissue preparation, immunofluorescence and quantitative analyses

After behavioral tests, mice were deeply anesthetized with 2% sodium pentobarbital (60 mg/kg, i.p.). The hippocampus was rapidly isolated on ice, fixed in the 4% paraformaldehyde for 2 h, and dehydrated in 30% sucrose at 4 °C overnight. The hippocampus was embedded in optimum cutting temperature compound, cut in 10-μm-thick sections on a freezing microtome, and mounted on glass slides. Slices were blocked with 1% bovine serum albumin for 1 h at room temperature followed by incubating with the following primary antibodies: rabbit anti-GFAP (1:600; Abcam), goat anti-DCX antibody (1: 200; Santa Cruz) and rabbit anti-Ki67 (1:500; Abcam) in 1% bovine serum albumin at 4 °C overnight. Subsequently, the sections were incubated with 1:200 FITC- or 1:400 Cy3- conjugated IgG (FITC and Cy3 conjugated, donkey anti-goat for Pirt; Cy3 conjugated, donkey anti-rabbit for P2 × 3; FITC conjugated, donkey anti-mouse for Tuj-1 and SMA) for 2 h to visualize target proteins. All staining procedures were carried out at room temperature, and all of the incubations were separated by three washes in PBS for 5 min each. Images were taken with a Nikon digital camera DXM1200 (Nikon, Japan) attached to a Nikon Eclipse E600 microscope (Nikon). For systematic random sampling in design-based cell counting, every third brain section was analyzed across the regions of interest. To count positive cells in the hippocampus, three fields per brain section were evaluated under 20X magnification of a light microscope and all immunopositive cells were counted. The same slide contained control and experimental sections. Counts are expressed as the average cell number per field ± standard error (SE) of means.

### Measurement of 5-HTcontents in hippocampus

Levels of 5-HT were detected by high-performance liquid chromatography (HPLC) as described^26^. Hippocampus tissues were homogenized in 200 μl of perchloric acid, then centrifuged at 12,000 rpm (4 °C) for 20 min. The supernatant was used for 5-HTdetection by HPLC. The mobile phase contained 85 mM citrate, 100 mM sodium acetate, 0.9 mM octyl-sodium sulfate, 0.2 mM EDTA and 12% methanol, pH 3.7. Twenty microliter of supernatant was injected through a system consisting of an LC-10A pump with a flow rate of 1.0 ml/min. External standard curves were used to quantify the amount of 5-HT in each sample according to the area under curve.

### UPLC-Q-TOF/MS analysis of potential metabolites in hippocampus

UPLC-Q-TOF/MS analysis was performed on an Agilent 1290 Infinity LC system equipped with an Agilent 6530 Accurate-Mass Quadrupole Time-of-Flight (QTOF) mass spectrometer (Agilent Technologies, CA). Chromatographic separations were performed at 40 °C on an ACQUITY UPLC HSS T3 column (2.1 mm × 100 mm, 1.8 μm, Waters, Milford, MA). The mobile phase consisted of 0.1% formic acid (A) and acetonitrile modified with 0.1% formic acid (B). The optimized UPLC elution conditions were 0–2 min, 5% B; 2– 10 min, 5–15% B; 10–14 min, 15–30% B; 14–17 min, 30–95% B; 17–19 min, 95% B, and the post time was set to 6 min for equilibrating the system. The flow rate was set to 0.4 ml/min and the injection volume was 3 μl. The auto-sampler was maintained at 4 °C. An electrospray ionization source (ESI) was used both in positive mode and negative mode. The optimized conditions were as follows: capillary voltage, 4 kV for positive mode and 3.5 kV for negative mode; drying gas flow, 11 L/min; gas temperature, 350 °C; nebulizer pressure, 45 psig Fragmentor voltage, 120 V; skimmer voltage, 60 V. Data were collected in centroid mode from 50 to 1,100 m/z. Potential biomarkers were further analyzed by MS/MS, and the collision energy was set to 10–50 eV.

### Identification of biomarkers

Potential biomarkers were identified through following steps. Firstly, confirm the ions based on the extracted ion chromatogram (EIC); secondly, input the accurate molecular mass of ions exact into online databases to search possible metabolites, such as Metlin (http://metlin.scripps.edu/), Human Metabolome Database (http://www.hmdb.ca/), and the Mass Bank (http://www.massbank.jp/); thirdly, compare the MS/MS spectra with the MS/MS information from databases in order to verify the structure the putative metabolites; lastly, the metabolites were identified using standard samples based on the retention time and the fragments information. A metabolic pathway was drawn by the Smartdraw software based on identified potential biomarkers (SmartDraw 7.5 Hemera Technologies Inc).

### Real-time RT-PCR

Total RNA from hippocampus was isolated using an Ultraspec RNA isolation kit (Biotecx). Real-time RT-PCR samples were run in triplicates on the ABI Prism 7000 sequence detection system. The primers for TNF-α, IL-1β, HMGB1, TLR4 and NF-κB are listed in Table 2. All real-time RT-PCR experiments were performed at least three times, and the mean ± SEM values are presented.

### Statistical analysis

Each sample was represented in electro spray ionization (ESI) chromatogram. The UPLC–MS raw data were converted in to a common data format (mzData) files by Agilent Mass Hunter Qualitative software, in which the isotope interferences were exclude with threshold set to 0.1%. The program XCMS (http://metlin.scripps.edu/download/) was applied for peak detection, RT alignment and peak integration to generate a visual data matrix. After filtering the ions based on 80% rule, the data of each sample was normalized to total intensity to correct the MS response shift. Then the three-dimensional data including RT m/z pair, sample name, and normalized ion intensity were imported to SIMCA-P software (version 11.0, Umetrics, Umea, Sweden) for principal component analysis (PCA) and partial least squares-discriminate analysis (PLS -DA). The heat map of the different metabolites was performed online by the MetaboAnalyst platform (http://www.metaboanalyst.ca).

Data are represented as mean ± SEM. The statistical significance of differences was analyzed using the SPSS 17.0 program (IBM, New York, USA). Statistical analyses were performed using a Student’s t-test and one-way ANOVA. For behavior responses, two-way ANOVA with repeated measure analyses of variance were performed followed by the Bonferroni test post hoc test for multiple comparisons. P < 0.05 was considered statistically significant.

## Discussion

Previous studies reported that LPS, at a dose of 5 mg/kg, could induce a long-lasting neuro-inflammation and showed persistent cognitive and behavioral abnormality in animals^24^,^27^ which resemble the symptoms of endotoxemia related encephalopathy in patients^4^,^22^,^23^,^24^. What is more, this finding was verified by metabolic analyses of hippocampus, indicating 5-HT related metabolic pathway was involved in the pathogenesis of endotoxemia induced acute neuro-inflammation and cognitive dysfunction. Our present study showed that endotoxemia induced acute neuro-inflammation is associated with the disruption of neurotransmitters, impairment of neuron genesis, and activation of astrocytes coupled with concomitant neuro-inflammation. By integrating different experimental platforms, we proved dioscin displays a positive regulatory effect on 5-HT levels in hippocampus as well as the activation of neuro-inflammatory cells and neurogenesis.

**Figure 10:**
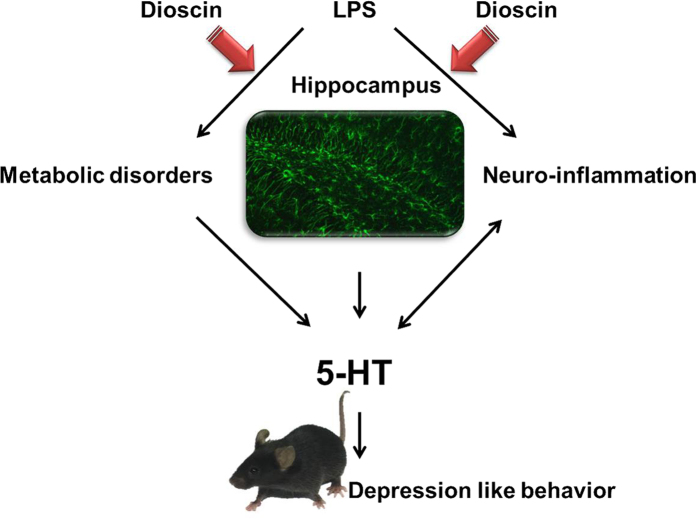
A schematic diagram of the experimental rationale for the design and results of our research.

For convenience, we had drawn a schematic diagram of the experimental rationale, shown in Fig. 10. During the experiment, we found that neuro-transmitter 5-HT played a key role in the pathological process, on one hand the change in the neuro-transmitter 5-HT content directly explained the change in the behavior of the mice; on the other hand by using metabolomics techniques, we found changes in hippocampal tissue metabolites and metabolic pathways, especially the change in tryptophan related metabolic pathway explains the hippocampus 5-HT content change. Meanwhile, previous studies have shown: tryptophan, including 5-HT, has a protective effect on neuro-inflammation^18^, so we speculate: classic HMGB1/TLR4 pathway related inflammation may be involved in endotoxemia induced acute neuro-inflammation pathological processes.

It has been proved that the IL-1 related pathways regulate inflammatory reactions, hematopoiesis and cognition, and sepsis related systemic inflammatory response is an important step in the development of cognitive dysfunction^28^,^29^,^30^,^31^,^32^. Previous studies have revealed that IL-1β, IL-6 and TNF-α are important inflammatory mediators in cognitive impairment^33^. In addition, the interaction between inflammatory mediators enhances endotoxemia induced cognitive dysfunction^33^, as the neuro-inflammatory cells in CNS, both microglia and astrocytes are activated after sepsis onset in the hippocampus^33^. Since one of the vital characteristics in neuro-inflammation is the production and release of inflammatory mediators from central immune cells like microglia and astrocyte^34^,^35^, the blockade of astrocyte activation by dioscin may lead to decreases in inflammatory mediators in the hippocampus.

Although physiological levels of cytokines, such asIL-1β, TNF-α, IL-6, and IFN-γ, are essential for synaptic plasticity and neurogenesis, the elevation of those cytokines will have damaging effects^36^,^37^. At high levels, IL-1β reduced hippocampal neurogenesis^38^, which is consistent with our immunofluorescence results (Fig. 5), showing that the number of DCX and Ki67 positive neurons in LPS treated group was greatly decreased compared with control group. All of these findings could be the mechanism for the persistence of neuro-functional deficits and impaired performance in the behavioral tests. Dioscin prevented the decreases in the number of DCX and Ki67 positive cells in hippocampus, indicating that dioscin has a neuro-protective effect on neurogenesis and neuron differentiation.

Although mechanisms underlying endotoxemia induced acute neuro-inflammation are complex and controversial, recent studies have proven that TLR4 signal pathway is in participation with endotoxemia related cognitive dysfunction^39^. The activation of TLR4 signal pathway started from recognizing particular TLR ligands including LPS and LPS/TLR4 signaling, has been proved to be involved in endotoxemia induced multiple organ dysfunction^40^. The combination of TLR4 and MyD88 activates the downstream high-mobility group protein 1 (HMGB-1)^41^,^42^,^43^. NF-κB, a critical factor in the regulation of this inflammatory response, stimulates the production and release of inflammatory mediators, such as interleukins (IL-1β and IL-6) and TNF-α, by inflammatory cells^44^,^45^,^46^,^47^. Based on the real-time RT-PCR results), the inflammatory regulatory capability of dioscin is partially dependent on the decreased levels of NF-κB, HMGB-1, TNF-α, IL-1β and IL-6, by reducing the TLR4 signaling. From these data, we inferred that dioscin could modulate neuro-inflammation responses through HMGB-1/TLR4 signaling pathway. However, it warrants further studies to demonstrate whether the production and release of TNF-α, IL-1βand IL-6 is altered in HMGB1/TLR4 signaling pathway and whether this is the main pathological mechanism in endotoxemia induced acute neuro-inflammation.

Overall, we demonstrated that the disruption of neurotransmitters, impairment of neurongenesis and activation of astrocytes coupled with concomitant neuro-inflammation via HMGB-1/TLR4 signaling pathway were the potential pathogenesis of cognitive impairment in sepsis survivors. Furthermore, the current findings suggested that dioscin could be a potential therapeutic approach for endotoxemia induced acute neuro-inflammation.

## Additional Information

**How to cite this article**: Yang, R. *et al*. Dioscin relieves endotoxemia induced acute neuro-inflammation and protect neurogenesis via improving 5-HT metabolism. *Sci. Rep.* **7**, 40035; doi: 10.1038/srep40035 (2017).

**Publisher's note:** Springer Nature remains neutral with regard to jurisdictional claims in published maps and institutional affiliations.

## Supplementary Material

Supplementary Figures

## Figures and Tables

**Table 1:** Identification of significantly differential metabolites for hippocampus from control, LPS treated and low dose of dioscin treated groups by UPLC-MS/TOF.

Related metabolic pathway	No.	MZ	Ion	Metabolites	Formula	LPS/Control		Dioscin/LPS	
						fold change	P value	fold change	P value
Arachidonic acid	1	353.2326	[M + H]+	PGH2	C20H32O5	1.1500	0.0016	1.2321	0.0012
	2	375.2143	[M + Na]+	PGE2	C20H32O5	0.8840	0.0027	1.6923	0.0000
Arginine and proline	3	154.0578	[M + Na]+	Creatine	C4H9N3O2	1.0802	0.0005	1.2793	0.0000
	4	228.0288	[M + H]+	L-Glutamyl 5-phosphate	C5H10NO7P	1.1193	0.0026	1.0912	0.0072
Cysteine and methionine	5	385.1289	[M + H]+	S-Adenosylhomocysteine	C14H20N6O5S	1.1974	0.0000	1.2141	0.0000
Glutathione	6	308.0914	[M + H]+	Glutathione	C10H17N3O6S	0.7764	0.0000	1.3914	0.0000
Glycerophospholipid	7	146.1167	[M + H]+	Acetylcholine	C7H15NO2	3.5316	0.0000	0.4585	0.0001
	8	184.0734	[M + H]+	Phosphocholine	C5H14NO4P	0.8385	0.0056	0.8516	0.0264
	9	468.3089	[M + H]+	LysoPC(14:0)	C22H46NO7P	1.2210	0.0000	1.1730	0.0000
	10	280.0914	[M + Na]+	sn-glycero-3-Phosphocholine	C8H20NO6P	0.8610	0.0000	2.0042	0.0000
Histidine	11	123.0554	[M + H]+	Niacinamide	C6H6N2O	1.0166	0.0108	1.2239	0.0000
	12	303.2322	[M + Na]+	Linoleic acid	C18H32O2	0.5490	0.0000	1.8566	0.0000
Pantothenate and CoA	13	113.0344	[M + H]+	Uracil	C4H4N2O2	0.7987	0.0000	1.2490	0.0000
	14	359.1035	[M + H]+	D-Pantetheine -phosphate	C11H23N2O7PS	0.8130	0.0000	1.4000	0.0000
	15	220.1184	[M + H]+	Pantothenic Acid	C9H17NO5	2.3396	0.0000	1.3168	0.0000
Phenylalanine	16	166.0862	[M + H]+	L-Phenylalanine	C9H11NO2	1.2370	0.0000	1.2683	0.0000
	17	135.07	[M + H]+	2-Phenylacetamide	C8H9NO	1.0796	0.0015	1.2073	0.0000
	18	194.0811	[M + H]+	Phenylacetylglycine	C10H11NO3	2.1954	0.0000	1.2893	0.0004
Purine	19	136.0619	[M + H]+	Adenine	C5H5N5	0.7372	0.0000	1.1436	0.0000
	20	137.0457	[M + H]+	Hypoxanthine	C5H4N4O	0.9736	0.0027	1.2761	0.0000
	21	152.0568	[M + H]+	Guanine	C5H5N5O	0.9606	0.0039	1.2176	0.0000
	22	153.041	[M + H]+	Xanthine	C5H4N4O2	0.5656	0.0000	0.7322	0.0118
	23	268.1045	[M + H]+	Adenosine	C10H13N5O4	0.7790	0.0000	0.8587	0.0003
	24	284.0983	[M + H]+	Guanosine	C10H13N5O5	1.2923	0.0004	0.7470	0.0002
	25	364.0653	[M + H]+	Guanidylic acid	C10H14N5O8P	1.2923	0.0004	0.7470	0.0002
	26	464.0816	[M + H]+	Adenylosuccinate	C14H18N5O11P	1.2415	0.0002	1.3354	0.0000
	27	243.0624	[M−H]−	Uridine	C9H12N2O6	0.8027	0.0000	1.2607	0.0000
Sphingolipid	28	300.2901	[M + H]+	Sphingosine	C18H37NO2	1.1731	0.0000	1.1112	0.0001
Steroid hormone	29	347.2216	[M + H]+	Corticosterone	C21H30O4	0.7257	0.0000	2.1854	0.0000
Tryptophan	30	138.0543	[M + H]+	Anthranilic acid	C7H7NO2	3.5316	0.0000	0.4585	0.0001
	31	160.0761	[M + H]+	Indoleacetaldehyde	C10H9NO	1.3305	0.0000	1.7536	0.0000
	32	177.1023	[M + H]+	Serotonin	C10H12N2O	1.3042	0.0000	1.7259	0.0000
	33	192.0658	[M + H]+	5-Hydroxyindoleacetic acid	C10H9NO3	1.6147	0.0000	1.5743	0.0000
	34	205.0975	[M + H]+	L-Tryptophan	C11H12N2O2	1.3103	0.0000	1.3775	0.0000

Potential biomarkers related to endotoxemia induced acute neuro-inflammation and the related metabolic pathways.

**Table 2:** The primer sequences used for real-time RT-PCR assay were listed as below.

Gene	Forward primer(5′-3′)	Reverse primer(5′-3′)
Mouse β-actin	ATGACCCAAGCCGAGAAGG	CGGCCAAGTCTTAGAGTTGTTG
Mouse TNF-α	GGAACACGTCGTGGGATAATG	GGCAGACTTTGGATGCTTCTT
Mouse IL-1β	ACCTGCTTTCCCCAAAACGAA	TGAGAGAAGTCGCACTGAGTC
Mouse HMGB-1	GGCGAGCATCCTGGCTTATC	GGCTGCTTGTCATCTGCTG
Mouse TLR-4	AGCTCCTGACCTTGGTCTTG	CGCAGGGGAACTCAATGAGG
Mouse NF-kb	ATGGCAGACGATGATCCCTAC	TGTTGACAGTGGTATTTCTGGTG
Mouse Myd88	TCATGTTCTCCATACCCTTGGT	AAACTGCGAGTGGGGTCAG
Human HMGB-1	TATGGCAAAAGCGGACAAGG	CTTCGCAACATCACCAATGGA
Human TLR4	AGACCTGTCCCTGAACCCTAT	CGATGGACTTCTAAACCAGCCA
Human Myd88	GGCTGCTCTCAACATGCGA	CTGTGTCCGCACGTTCAAGA

The primer sequences used for real-time RT-PCR.
