# Supplementary material for: Dioscin relieves endotoxemia induced acute neuro-inflammation and protect neurogenesis via improving 5-HT metabolism
Source: Sci Rep. 2017 Jan 6;7:40035. doi: 10.1038/srep40035 (PMC5216397; doi:10.1038/srep40035)

Dioscin relieves endotoxemia induced acute neuro-inflammation and protect neurogenesis via improving 5-HT metabolism

Rui Yang, Wei Chen, Ye Lu, Yingke Li, Hongli Du, Songyan Gao, Xin Dong, Hongbin Yuan

Supplementary materials

Figure legends

Fig. S1.Analysis of cell proliferation of primary peritoneal macrophages (PM) RAW 264.7 ThP1 and U251 cell lines after 24 hours dioscin treatments at different concentrations of (50, 100, 200 ng/ml). There were no significant differences between dioscin treated group and control group, which indicated dioscin did not have an effect on cell proliferation even at high concentrations.

Fig.S1

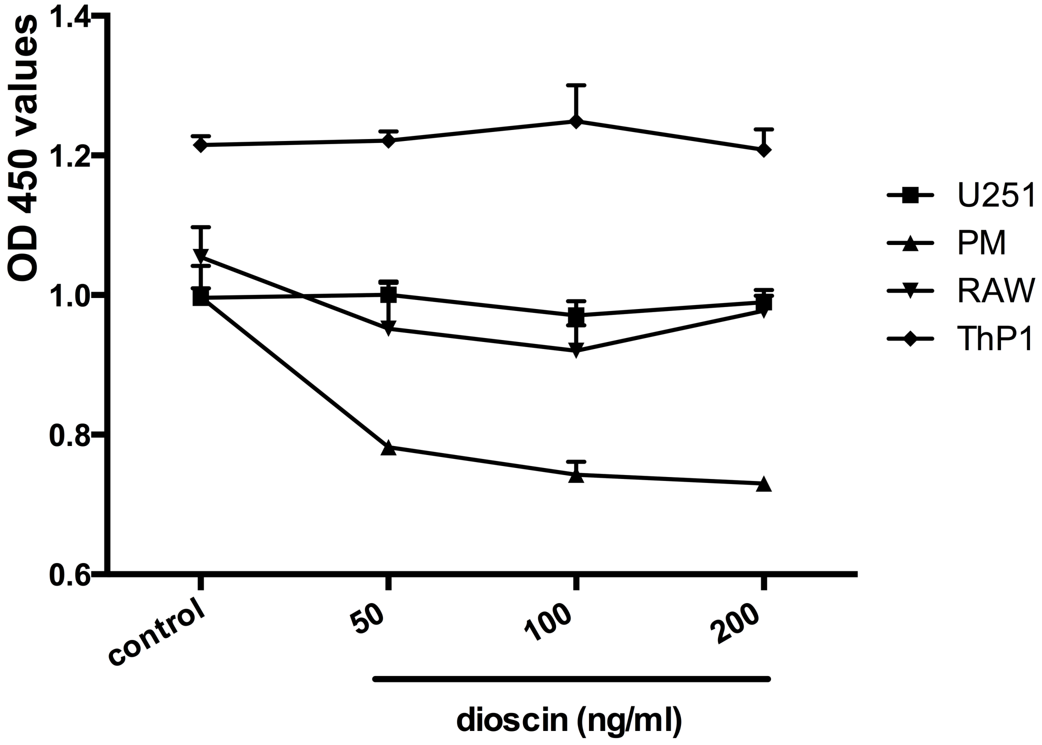

Fig. S2. Annexin-V/propidium iodide (PI) double staining of apoptosis analysis was conducted by using flow cytometry. Different cell lines including primary peritoneal macrophages (PM) RAW 264.7 ThP1 and U251 cell lines were treated with a blank control and different concentrations of dioscin (50, 100, 200 ng/ml) for 24 hours

before double-stained with Annexin-V and PI. Apoptotic percentage (%) was calculated as the sum of early-stage apoptosis and late-stage apoptosis. There were no scientific differences between dioscin treated group and control group, which indicated dioscin did not induce cell apoptosis even at high concentrations.

Fig.S2

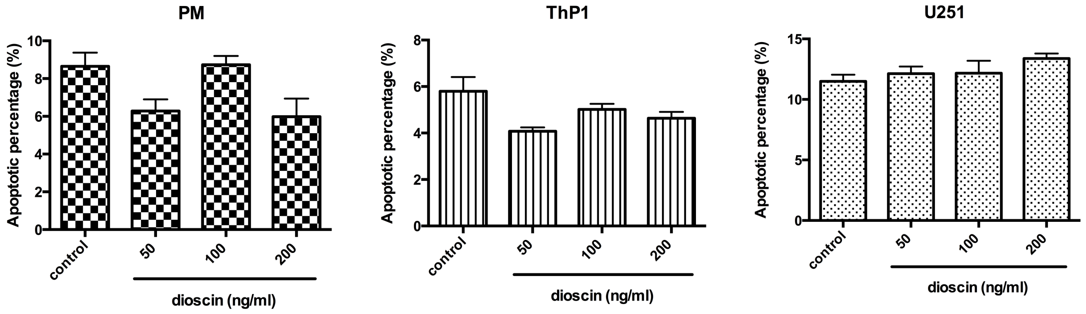

Supplement: Supplementary Figures [file srep40035-s1.pdf]
